# Emergence and Genomic Characterization of the First Reported *optrA*-Carrying Linezolid-Resistant Enterococci Isolated from Retail Broiler Meat in the United Arab Emirates

**DOI:** 10.3390/foods11203190

**Published:** 2022-10-13

**Authors:** Ihab Habib, Akela Ghazawi, Glindya Bhagya Lakshmi, Mohamed-Yousif Ibrahim Mohamed, Dan Li, Mushtaq Khan, Shafi Sahibzada

**Affiliations:** 1 Veterinary Public Health Research Laboratory, Department of Veterinary Medicine, College of Agriculture and Veterinary Medicine, United Arab of Emirates University, Al Ain 52571, United Arab Emirates; 2 Department of Environmental Health, High Institute of Public Health, Alexandria University, Alexandria 5424041, Egypt; 3 Department of Medical Microbiology, College of Medicine and Health Sciences, United Arab Emirates University, Al Ain 52571, United Arab Emirates; 4 Department of Food Science and Technology, National University of Singapore, Singapore 119077, Singapore; 5 Antimicrobial Resistance and Infectious Diseases Laboratory, Murdoch University, Murdoch, WA 6150, Australia

**Keywords:** enterococci, poultry, antimicrobial resistance, whole-genome sequencing, UAE

## Abstract

The foodborne transfer of resistant genes from enterococci to humans and their tolerance to several commonly used antimicrobials are of growing concern worldwide. Linezolid is a last-line drug for managing complicated illnesses resulting from multidrug-resistant Gram-positive bacteria. The *optrA* gene has been reported in enterococci as one of the acquired linezolid resistance mechanisms. The present study uses whole-genome sequencing analysis to characterize the first reported isolates of linezolid-resistant *E. faecium* (n = 6) and *E. faecalis* (n = 10) harboring the *optrA* gene isolated from samples of supermarket broiler meat (n = 165) in the United Arab Emirates (UAE). The sequenced genomes were used to appraise the study isolates’ genetic relatedness, antimicrobial resistance determinants, and virulence traits. All 16 isolates carrying the *optrA* gene demonstrated multidrug-resistance profiles. Genome-based relatedness classified the isolates into five clusters that were independent of the isolate sources. The most frequently known genotype among the isolates was the sequence type ST476 among *E. faecalis* (50% (5/10)). The study isolates revealed five novel sequence types. Antimicrobial resistance genes (ranging from 5 to 13) were found among all isolates that conferred resistance against 6 to 11 different classes of antimicrobials. Sixteen different virulence genes were found distributed across the *optrA*-carrying *E. faecalis* isolates. The virulence genes in *E. faecalis* included genes encoding invasion, cell adhesion, sex pheromones, aggregation, toxins production, the formation of biofilms, immunity, antiphagocytic activity, proteases, and the production of cytolysin. This study presented the first description and in-depth genomic characterization of the *optrA*-gene-carrying linezolid-resistant enterococci from retail broiler meat in the UAE and the Middle East. Our results call for further monitoring of the emergence of linezolid resistance at the retail and farm levels. These findings elaborate on the importance of adopting a One Health surveillance approach involving enterococci as a prospective bacterial indicator for antimicrobial resistance spread at the human–food interface.

## 1. Introduction

Enterococci are Gram-positive bacteria commonly present in several environments, including humans’ and animals’ gastrointestinal microbiota, and they are considered a potential opportunistic pathogen responsible for healthcare-associated infections [1]. The most significant share of enterococcal infections is caused by *Enterococcus faecalis* and *Enterococcus faecium*. These two species are also known for their potential for transferring and acquiring genes conferring antimicrobial resistance from the same or different species [2], for instance, through passing mobile genetic elements [3]. In the food chain, the acquisition of resistant genes from enterococci of animal origin by humans and their tolerance to several commonly used antimicrobials are of growing concern worldwide [2,3]. There are limited treatment options for enterococcal infections since the organism demonstrates an intrinsic resistance to antimicrobials such as cephalosporins, polymyxin, colistin, sulfonamides, and small concentrations of aminoglycosides [4]. Thus, monitoring antimicrobial resistance patterns in enterococci in the food chain has been recommended in different countries.

Linezolid, the first clinically available oxazolidinone class of antibiotics, acts in bacteria by binding the 23S rRNA component (in the V domain) of the 50S ribosomal unit and disrupts the synthesis of protein. Linezolid is a last-line drug to treat complicated outcomes from multiclass-resistant Gram-positive bacteria, such as vancomycin-resistant *Enterococcus* spp. and *Staphylococcus aureus* exhibiting methicillin resistance [5]. Among the acquired (mobile) linezolid resistance mechanisms, the *optrA* gene has been reported as being responsible for the recent emergence in linezolid-resistant enterococci among isolates from human clinical sources [6]. The *optrA* gene exists mainly in plasmids and is responsible for phenicols resistance (florfenicol and chloramphenicol) and resistance to oxazolidinones (tedizolid and linezolid) [7]. Even though linezolid is not permitted in the livestock industry, linezolid-resistant *E. faecalis* and *E. faecium* isolates have been detected in several countries in livestock and food of animal origin [6,7]. Recently, we reported, for the first time in the United Arab Emirates (UAE), the resistance of *E. faecalis* and *E. faecium* to linezolid harboring the *optrA* gene in isolates recovered from supermarket chicken carcasses [8].

Whole-genome sequencing (WGS) is a promising tool that provides information at the single nucleotide level and can be used to gain better insight into the antimicrobial resistance of multidrug-resistant bacteria [9]. The present study uses WGS to characterize the first reported isolates of linezolid-resistant *E. faecalis* and *E. faecium* harboring the *optrA* gene from retail chicken meat in the UAE. The sequenced genomes in the current study were used to gain insight into the genetic relatedness of the studied isolates, their antimicrobial resistance determinants and virulence traits, and other molecular epidemiological features. This work highlights the importance of monitoring resistance to clinically viable antimicrobials, such as linezolid, with the One Health approach. The genomic insight gained from this investigation adds to the local and global knowledge on the molecular epidemiology of emerging antimicrobial resistance at the human–food interface.

## 2. Materials and Methods

### 2.1. Study Setting and Chicken Samples

The linezolid-resistant *E. faecalis* and *E. faecium* harboring the *optrA* gene included in this research were recovered within a baseline survey of retail chicken carcasses in Abu Dhabi Emirate, the UAE. The details of sampling and the full results of the baseline survey are published elsewhere [8]. In summary, 165 chilled whole chicken carcasses were collected from supermarket chains. The carcasses belonged to seven different poultry processing companies (brands), of which six are UAE processors, and one originated from the Kingdom of Saudi Arabia. All broiler carcasses were collected from the refrigerated displays presented at several retail markets [8].

### 2.2. Isolates Identification and Screening for Antimicrobial Resistance

According to previously published methods, the recovery of enterococci from retail chicken carcasses was achieved [10]. Species verification was completed using the Vitek-2 system with Vitek-2 GP-ID cards (bioMérieux, Marcy l’Etoile, France). *Enterococcus* spp. resistance to ten antimicrobials was evaluated using a Vitek AST-P592 panel (bioMérieux) that tested the specified species (*E. faecalis*/*E. faecium*) for the following antimicrobials: ciprofloxacin, erythromycin, ampicillin, high-level gentamicin, high-level streptomycin, vancomycin, teicoplanin, tigecycline, tetracycline, and linezolid. According to the manufacturer’s instructions, the isolates were considered susceptible, intermediate, or resistant [8]. The CLSI guidelines were used to classify isolates as resistant and susceptible. The multidrug resistance profile was denoted by resistance to at least one antimicrobial in three or more antimicrobial classes [11]. *E. faecium* ATCC29212 was used as a control strain.

Among the 16 linezolid-resistant *Enterococcus* (LRE) isolates (MIC (minimum inhibitory concentration) of ≥8.0 mg/L), as well as among a selection (n = 20) of the linezolid-positive *Enterococcus* strains, the presence or absence of the *optrA*, *poxtA*, and *cfr* genes was evaluated using a multiplex PCR, according to Egan et al. [12].

### 2.3. Whole-Genome Sequencing Analysis

The extraction of DNA from the studied isolates was completed using a commercial kit (Wizard^®^ (Promega, Madison, WI, USA)). WGS was performed on an Illumina NovaSeq platform PE150 utilizing a send-out commercial service by the company Novogene in the United Kingdom. Using Spades version 3.14.0, the reads of the raw sequences were assembled into contigs (https://cge.cbs.dtu.dk/services/SPAdes/ (accessed on 15 May 2022)). The assembled contigs were uploaded to the online pipeline of Pathogenwatch (https://pathogen.watch (accessed on 20 May 2022) to achieve species confirmation. The genomes that exhibited a satisfactory quality were kept and utilized to determine in silico the sequence type (ST) using the MLST (multilocus sequence types) online tool (https://github.com/tseemann/mlst (accessed on 20 May 2022)). Genes conferring resistance were detected using Antimicrobial Resistance Gene Finder (AMRFinder; https://github.com/ncbi/amr; via NCBI (accessed on 25 May 2022)), and the virulence factors were predicted via ABRicate V1.0.1 (https://github.com/tseemann/abricate (accessed on 28 May 2022)) using the NCBI’s Resfinder and the universal virulence finder database VFDB. A cut-off value of 98% was used for gene coverage and identity.

A phylogenetic tree (approximate maximum-likelihood) was constructed on single nucleotide polymorphisms (SNPs) in the core genomes of the studied isolates that were aligned using the online tool SNIPPY V4.1.0 (https://github.com/tseemann/snippy (accessed on 28 May 2022)). Following the removal of recombinants with ClonalframeML v1.12 (https://github.com/xavierdidelot/ClonalFrameML/releases (accessed on 15 June 2022)), SNP alignment was used to produce a maximum-likelihood tree utilizing RAxML-NG (V1.1.0) (https://github.com/amkozlov/raxml-ng/releases (accessed on 15 June 2022)). The relationship between the studied isolates was established by a pan-genome analysis using gene content; this was maintained using the Roary (v3.20.0) pan-genome pipeline with default parameters (https://sanger-pathogens.github.io/Roary/ (accessed on 20 June 2022)). The Roary analysis aided in determining the number of accessory and core genes present in the genomes of all isolates.

### 2.4. Genome Sequence Data Availability

All WGS assemblies related to this study have been deposited in the NCBI database under BioProject ID: PRJNA850926. New sequences of the seven housekeeping genes were submitted.

## 3. Results

### 3.1. Phenotypic and PCR-Based Confirmation of Linezolid Resistance

Seventeen isolates were phenotypically resistant to linezolid (MIC of ≥ 8.0 mg/L). Of these isolates, 11 (64.7%) were *E. faecalis,* and 6 (35.3%) were *E. faecium* (Table 1). The linezolid-resistant isolates were traced back to three different processors, of which ten were from samples of company A (*E. faecalis* (n = 6) and *E. faecium* (n = 4)) and six were from company G (*E. faecalis* (n = 4) and *E. faecium* (n = 2)) (Table 1).

The PCR of the linezolid-resistant genes revealed the *optrA* gene’s amplification in all seventeen isolates, while the *cfr* gene was not present. Both the *optrA* and *poxtA* genes were amplified together in one *E. faecium* isolate. All isolates carrying the *optrA* gene demonstrated a multidrug-resistance (MDR) profile (Table 1). All of the 17 MDR-*optrA*-carrying *Enterococci* were submitted to WGS. However, one (*E. faecalis* isolate) out of the seventeen failed quality control checks for reads assembly was classified as ‘rejected.’ Hence, sixteen isolates were retained for further genomic analysis.

### 3.2. Population Structure and Clonal Relationship

**Figure 1:**
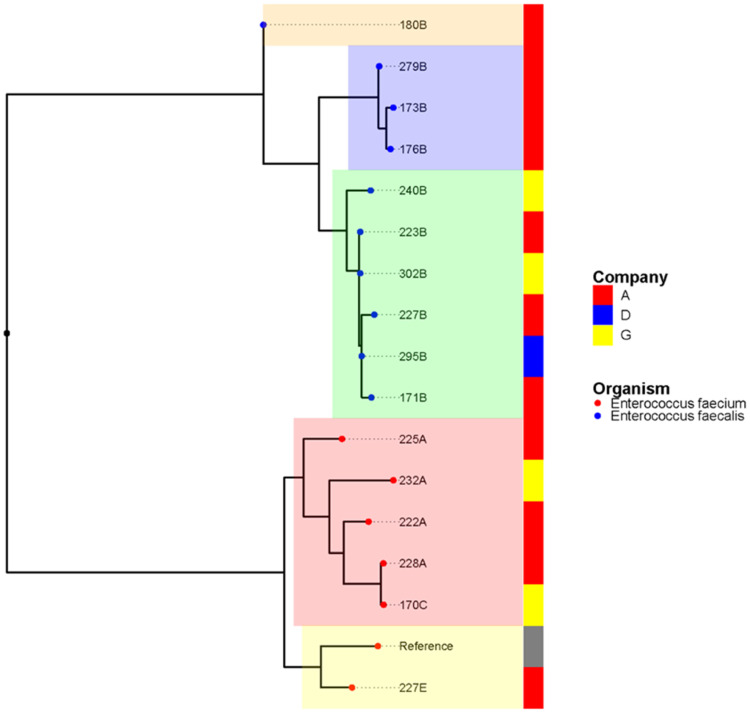
Maximum-likelihood phylogenetic rectangular tree based on single nucleotide polymorphisms (SNP). The tree contains 10 *E. faecalis* (blue tip color) and 6 *E. faecium* (red tip color) isolates from retail chicken meat collected in the UAE. The species influenced the clustering among the isolates, with two clusters identified in *E. faecium* and three in *E. faecalis*. No clustering was found based on the processing companies (A, D, G).

**Figure 2:**
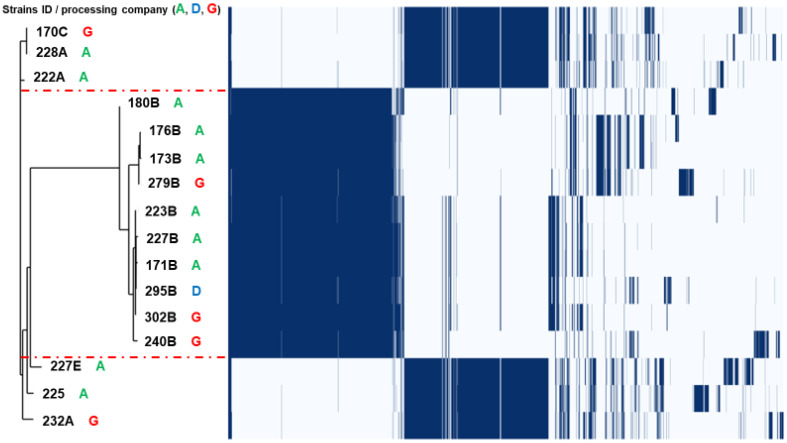
Pan-genome genetic relatedness analysis (utilizing the Roary (v3.20.0) pan-genome pipeline) of sixteen *optrA*-carrying linezolid-resistant *Enterococcus* spp. strains from retail chicken carcasses (from three processing companies: A, D, and G). Clustering was completed according to strains profiles by gene content. A total of 7651 genes were identified. Left: core-genome phylogeny; the three *E. faecalis* clusters (10 isolates) in the dendrogram are delineated by red dotted lines. Right: heatmap of the core genes. Each row shows the gene profile of each isolate (gene presence (blue)/absence (white) matrix from the pan-genome analysis).

An SNP-based maximum-likelihood phylogenetic tree (Figure 1) showed the discriminatory results within the species, with more diversity found among the *E. faecalis* than *E. faecium* isolates. The core genome tree identified five clusters, with three in *E. faecalis* and two in *E. faecium* that were independent of the isolate sources (Figure 1). There was no correlation between the isolate clusters and the origins (chicken processing companies) of the isolates, as similar clusters were found across different companies (Figure 1). The Roary matrix-based gene sequence analysis also revealed a similar pattern across the 16 isolates, with different clusters identified among the same companies using the total gene content of 7651 genes. The core genome analysis was very discriminatory within the species (*E. faecalis* versus *E. faecium*) and identified five clusters: three in *E. faecalis* and two in *E. faecium*, all of which were independent of the isolate sources (Figure 2).

The MLST analysis of the retained 16 isolates harboring the *optrA* gene revealed four known STs: three that were previously described for *E. faecalis* (ST476, ST1184, and ST314) and one for *E. faecium* (ST195), among which the most frequently known was ST476 (among 50% of linezolid-resistant *E. faecalis* (5/10)) (Table 2). Five novel sequence types have been assigned to the isolates characterized in this study, of which three STs were among *E. faecium* (ST2236, ST2238, and ST2239) and two were among *E. faecalis* (ST1290 and ST1291). According to the PubMLST database, all of the sequence types revealed in the studied isolates were unrelated to any known clonal complex (CC).

### 3.3. Genomic Analysis of Antimicrobial Resistance

Antimicrobial resistance genes (ranging from 5 to 13) were found among all the isolates that conferred resistance against 6 to 11 different classes of antimicrobials. All isolates carried resistance genes against the antimicrobial classes aminoglycosides, macrolides, phenicol, and oxazolidines (Table 2). Concerning the putative antimicrobial resistance genes identified, the WGS data supported the initial screening completed by the multiplex PCR detection of the *optrA* gene in all the linezolid resistant isolates and the concurrent detection of the *poxtA* and *optrA* genes in one isolate (*E. faecium* 227E). The PlasmidFinder results revealed the presence of ten different plasmid-associated replication genes. In only one *E. faecium* isolate, the *optrA* gene was associated with mobile genetic elements (the *repUS1* plasmid replicon) (Table 2). As presented in Table 2, the *fexA* gene conferring phenicol resistance was identified in all isolates. For genes conferring macrolide resistance, *erm(B)* and *lsa(A)* were in silico detected in all *E. faecalis* isolates, while the *msr(C)* gene (macrolide efflux pump) was identified only in *E. faecium* (and none in *E. faecalis*) (Table 2). All 16 isolates harbored several genes conferring resistance to high-level aminoglycosides, such as *aph(3’)-lll*. For genes conferring resistance to tetracycline, *tet(L)* was inferred in 14 of the 16 isolates (Table 2). Concerning genes conferring phenicol resistance, the *fexA* gene was identified in all isolates, and the *cat* gene was frequent among *E. faecalis* strains but not in *E. faecium* strains. In one of the strains (strain 222A (*E. faecium*)), the *optrA* gene was co-localized with both *erm(A)* (gene conferring erythromycin resistance) and *fexA* (gene mediating resistance to florfenicol) (Table 2). On the other hand, the *fexB* gene was presented in only one *E. faecium* strain, as was the *fosB3* gene, which confers fosfomycin resistance, and which was present in one *E. faecalis* strain (Table 2).

Aside from the resistance genes, the WGS analysis revealed the presence of the following four-point mutations in both species: *gyrA* (S83I and S83Y)*, parC* (S80I), and *eatA* (T450I) (Table 2). None of these point mutations are associated with linezolid resistance, and they are potentially linked to quinolone resistance. Of these four mutations, *parC* (S80I) was the most frequent and was identified in ten of the sixteen isolates. The WGS analysis of the studied isolates revealed the absence of ribosomal mutations associated with resistance to linezolid.

### 3.4. Genomic Insight into Virulence Traits

Based on the WGS data analysis, 16 virulence genes were found distributed across the *optrA*-carrying *E. faecalis* isolates (Table 3). The virulence genes in *E. faecalis* included genes encoding invasion, cell adhesion, sex pheromones, aggregation, toxins production, proteases, the formation of biofilms, antiphagocytic activity, immunity, and the production of cytolysin (Table 3). On the other hand, only one virulence gene (*acm*, associated with adhesins) was presented in the sequenced *E. faecium*.

## 4. Discussion

*Enterococcus faecalis* and *Enterococcus faecium* are the most frequently detected *Enterococcus* species in clinical and food samples, and these two species have a significant role in nosocomial infections in humans [2]. In the present study, we provide in-depth insight into *Enterococcus* isolates showing non-susceptibility to linezolid (MIC of ≥8.0 mg/L), one of the last to harbor antimicrobials for use against infection with Gram-positive bacteria exhibiting multidrug-resistance. This study is the first published genomic characterization of the *optrA* gene-harboring *E. faecium* and *E. faecalis* recovered from chicken meat for consumer retail in the Middle East.

### 4.1. A Concern for the Chicken Industry and Human Health

In this investigation, *optrA*-carrying linezolid-resistant isolates were presented in isolates belonging to three different chicken processors, which is, indeed, concerning. The *OptrA* gene has been found on plasmids (mobile elements) and is reported to be transmissible. Such a gene also has been shown to confer resistance to other important classes of antimicrobials, such as phenicols, macrolide–lincosamide–streptogramin B, and aminoglycoside [6]. Based on the WGS analysis presented in this investigation, we reported the presence of the *repUS1* plasmid among the characterized *Enterococci* isolates, and this plasmid is described in other studies as a possible *optrA* resistance carrier [13]. Aside from the potential spread of linezolid resistance through mobile genetic elements, acquired resistance to linezolid has been linked to livestock, where the utilization of phenicol and macrolides (linked to *optrA*) is hypothesized to co-select resistance to different antibiotic classes [14,15]. The co-selection of resistance may be a likely scenario, as could be inferred from the WGS analysis of the studied isolates. In one of the strains (strain 222A (*E. faecium*)), the *optrA* gene was co-presented with both *fexA* (conferring resistance to florfenicol) and *erm(A)* (mediating resistance to erythromycin). Such co-existence may indicate that the linezolid resistance in some strains generated from retail chicken meat may be co-selected due to the excessive use of non-oxazolidinone antimicrobials (such as florfenicol and macrolides). Collectively, these results call for further monitoring of the emergence of linezolid resistance at the retail and farm levels.

### 4.2. Clonal Relationship from a One Health Perspective

The SNP-based maximum-likelihood phylogenetic tree and pan-genome relatedness analysis of the 16 sequenced isolates revealed the variable composition gene contents that delineate the strains in five groups, but without evidence of a correlation between the isolate clades and the three chicken processing companies from which the isolates originated. This highlights the complexity and the dynamics of linezolid resistance spread in some of the UAE poultry processors, and it calls for further field epidemiological studies to understand what are, if any, the shared inputs and features between those three specific companies. Moreover, further analysis is required to understand the genomic plasticity in this isolate collection due to differences in the gene contents and other particular genomic features of the studied strains.

The MLST analysis identified ST476 as the most-known sequence type frequently detected in *E. faecalis* isolates. *OptrA*-positive ST476 linezolid-resistant strains have been reported in several countries, such as Korea, China, Malaysia, Italy, and Tunisia, and they have been frequently isolated from humans and livestock (chicken and pigs) [16]. A comparative genomics study that looked at the global dissemination of *optrA*-carrying *E. faecalis* found genetic relatedness between the *optrA*-positive *E. faecalis* of ST476 (and ST21) in animal and clinical (human) hosts from several regions of the world over several decades, which is a remarkable finding and demonstrates that they may originate from an animal reservoir and can efficiently colonize humans [16]. Next to ST476, two linezolid-resistant *E. faecalis* isolates were assigned as ST1184. Intestinally, the PubMLST database (accessed on 3 September 2022) currently holds only one record for *E. faecalis* isolated from China (assigned as ST1184), which was from the fecal specimen of a camel. Knowing that in the UAE, the camel is an important animal that is widely raised, owned, and domesticated for meat, dairy, and racing purposes, it is worth investigating the antimicrobial resistance patterns in the *Enterococci* population from camels and its potential role in the environmental spread of antimicrobial resistance. From a One Health point of view, these findings collectively call for the extensive surveillance of enterococci as indicators for antimicrobial resistance emergence.

### 4.3. Genomic Insight into Multidrug Resistance and Virulence Traits

Enterococci can facilitate the mobilization of resistance genes horizontally to other bacteria, such as *Listeria* spp., *Staphylococcus aureus*, and *Escherichia coli* [17]. The gene *tet(L)*, which encodes for an efflux pump, is presented as the most dominant tetracycline resistance gene in the isolates characterized in this study. The *tet(L)* gene was co-presented in several of these strains with the plasmid *repUS43*. Previous findings have reported enterococci (in chicken sources) harboring these genes in tetracycline-resistant strains [18,19]. Macrolides resistance among the studied isolates was mainly associated with the genes *erm(B)* and *erm(A)* among *E. faecalis*. The *erm(B)* and *erm(A)* genes encode a ribosomal methylase and are referred to as the critical genes in enterococci conferring erythromycin resistance, and the methylase could also switch resistance to streptogramin B and lincosamides [20]. The *msr(C*) gene that encodes an efflux pump was only presented in the *E. faecium* characterized in this study, which is in line with previous studies that have pointed out that the gene is likely to be essential for the *E. faecium* species [21].

Some linezolid-resistant isolates have exhibited high-level resistance to gentamicin (HLGR) and streptomycin, which are clinically essential antimicrobials in some complicated cases of enterococcus infection in humans [22]. Moreover, our WGS analysis noted the presence of acquired aminoglycoside resistance genes, which have been proven to confer high-level resistance to aminoglycosides [23]. Previous studies have reported the foodborne transmission of HLGR enterococci from livestock to humans [24]. WGS adds value to the current traditional approaches used in food and clinical microbiology, and it offers a new frontier to help with predicting antimicrobial resistance.

The present study noted a high frequency of virulence genes (n = 16) in *optrA*-carrying linezolid-resistant *E. faecalis*, which aligns with several studies showing, in general, a higher frequency of virulence genes in *E. faecalis* than in *E. faecium* of food origin [25,26]. The genes *gelE* and *fsrB* have been known to present concurrently in *E. faecalis* strains isolated from both sick and healthy animals [27]. The gelE gene and other virulence genes regulate the expression of the *fsr* operon [28]. The *gelE* encodes zinc endopeptidase extracellularly, targeting many substrates, such as gelatin and collagen. It emphasizes the pathogenesis of endocarditis induced by *E. faecalis* [29]. Enterococcal cytolytic toxin-encoding genes (*cylB, cylL,* and *cylM*) were found in one of the *E. faecali* isolates, which is interesting because cytolysin activities have been shown to facilitate the virulence of enterococci in infection models, and it has been linked with mortality consequences in some patients [30]. The co-presence of virulence genes and antimicrobial resistance determinants in *E. faecalis* may lead to a higher risk to human health upon the consumption of improperly cooked meat [31].

## 5. Conclusions

This study presents the first description and in-depth genomic characterization of *optrA*-gene-carrying linezolid-resistant enterococci from retail broiler meat in the UAE and the Arab world. The *Enterococcus* isolates characterized in this investigation showed a rich portfolio of genes conferring antimicrobial resistance and virulence factors. Our study calls for future research that involves the characterization of enterococci based on a more significant number of samples of chicken meat in order to add more power to this study’s findings. National monitoring of antimicrobial resistance in the UAE should further consider the matter of the emergence of linezolid- and multiclass-resistant enterococci in the chicken meat supply chain and its environments. For epidemiological surveillance, WGS is a valuable tool for collectively studying the resistome, virulome, and mobilome and identifying the spread of genotypes with potential clinical relevance from retail chicken to consumers. This investigation adds to the UAE’s (and to the global) understanding of the molecular epidemiology of antimicrobial resistance in poultry meat.

## Figures and Tables

**Table 1:** Resistance patterns of the seventeen *optrA*-carrying linezolid-resistant *Enterococcus* spp. strains from retail chicken carcasses.

Species	Strains	Company	Antimicrobial Resistance Phenotype *
*Enterococcus faecalis*	171B	A	CIP-ERYTH-LIN-TET
	223B	A	CIP-ERYTH-LIN
	227B	A	CIP-ERYTH-LIN-TET
	173B	A	CIP-ERYTH-LIN-TET
	176B	A	CIP-ERYTH-LIN-TET
	180B	A	HLS-CIP-ERYTH-LIN-TET
	295B	D	HLG-CIP-ERYTH-LIN-TET
	279B	G	HLG-CIP-ERYTH-LIN-TET
	302B	G	HLG-HLS-CIP-ERYTH-LIN-TET
	240B	G	HLG-HLS-CIP-ERYTH-LIN-TET
	288B **	G	HLG-CIP-ERYTH-LIN-TET
*Enterococcus faecium*	227E	A	HLG-CIP-ERYTH-LIN-TET
	222A	A	HLG-HLS-CIP-ERYTH-LIN-TET
	225A	A	ERYTH-LIN-TET
	228A	A	HLS-CIP-ERYTH-LIN-TET
	170C	G	HLG-HLS-CIP-ERYTH-LIN-TET
	232A	G	HLG-HLS-CIP-ERYTH-LIN-TET

* Abbreviations (and antimicrobial classes): CIP, ciprofloxacin (fluoroquinolones); ERYTH, erythromycin (macrolides); LIN, linezolid (oxazolidinones); TET, tetracycline (tetracyclines); HLG, high-level gentamicin (aminoglycosides); and HLS, high-level streptomycin (aminoglycosides). ** Strain 288B was not included in the whole-genome sequencing data analysis.

**Table 2:** Distribution of the multilocus sequence types (STs) and the clonal complexes (CCs), antimicrobial resistance determinants, and mobile genetic elements (MGE) in the sixteen *optrA*-carrying linezolid-resistant *Enterococcus* spp. strains from retail chicken carcasses.

Species	Strains	ST	Quinolone Point Mutation	Antimicrobial Resistance Genes *							Plasmid Replicon Type	MGEs Associated with ARGs
				Linezolid	Lincosamide	Aminoglycoside	Macrolide	Tetracycline	Trimethoprim	Phenicol		
*Enterococcus faecalis*	171B	476	*gyrA_S83I* and *parC_S80I*	*optrA*	*-*	*ant(9)-la* and *aph(3’)-lll*	*erm(A), lsa(A),* and *erm(B)*	*tet(L)*	*-*	*cat* and *fexA*	*-*	*-*
	223B	476		*optrA*	*-*	*ant(9)-la*	*erm(A), Isa(A),* and *erm(B)*	*-*	*-*	*fexA*	*-*	*-*
	227B	476	*gyrA_S83I* and *parC_S80I*	*optrA*	*-*	*ant(9)-la* and *aph(3’)-lll*	*erm(A), lsa(A),* and *erm(B)*	*tet(L)*	*dfrG*	*cat* and *fexA*	*rep9a*	*-*
	173B	1184	*gyrA_S83I* and *parC_S80I*	*optrA*	*lnu(G)*	*aph(3’)-lll*	*Isa(A), erm(A),* and *erm(B)*	*tet(L)*	*dfrG*	*cat* and *fexA*	*rep9a* and *repUS43*	*tet(L), erm(B), aph(3’)-lll,* and *cat on repUS43*
	176B	1184	*gyrA_S83I* and *parC_S80I*	*optrA*	*lnu(G)*	*aph(3’)-lll*	*Isa(A), erm(A),* and *erm(B)*	*tet(L)*	*dfrG*	*cat* and *fexA*	*rep9a* and *repUS43*	*tet(L), erm(B), aph(3’)-lll,* and *cat on repUS43*
	180B	1291 *	*gyrA_S83Y* and *parC_S80I*	*optrA*	*lnu(G)*	*aph(3’)-lll* and *ant(6)-la*	*Isa(A)* and *erm(B)*	*tet(L)*	*-*	*fexA*	*rep6, rep1, rep9b,* and *repUS43*	*tet(L) on repUS43--- aph(3’)-lll,* and *ant(6)-la on rep1*
	295B	476	*gyrA_S83I* and *parC_S80I*	*optrA*	*-*	*aph(3’)-lll, ant(9)-la,* and *aac(6’)-aph(2’’)*	*erm(A), erm(B),* and *lsa(A)*	*tet(L)*	*-*	*fexA*	*repUS11* and *rep9b*	*-*
	279B	314	*eatA_T450I*	*optrA*	*lnu(G)*	*aph(3’)-lll* and *aac(6’)-aph(2’’)*	*erm(A), erm(B),* and *lsa(A)*	*tet(L)*	*-*	*cat* and *fexA*	*repUS11, repUS43,* and *rep9a*	*tet(L), erm(B),* and *cat on repUS43*
	302B	476	*gyrA_S83Y* and *parC_S80I*	*optrA*	*lnu(B)*	*aph(3’)-lll, ant(9)-la,* and *aac(6’)-aph(2’’)*	*erm(A), erm(B), Isa(A),* and *Isa(E)*	*tet(L)*	*dfrG*	*cat* and *fexA*	*repUS43*	*tet(L) on repUS43*
	240B	1290 *	*eatA_T450I*	*optrA*	*lnu(B)*	*aph(3’)-lll, ant(9)-la,* and *aac(6’)-aph(2’’)*	*erm(A), erm(B), lsa(A),* and *lsa(E)*	*tet(L)*	*dfrG*	*fexA*	*repUS11, rep9a,* and *rep6*	*-*
*Enterococcus faecium*	227E	195		*optrA, poxtA*	*lnu(G)*	*aac(6’)-li* and *aadD*	*erm(A), msr(C),* and *erm(B)*	*tet(L)*	*dfrG*	*fexA* and *fexB*	*rep2, rep14a, repUS15,* and *rep29*	*-*
	222A	2236 *	*gyrA_S83Y* and *parC_S80I*	*optrA*	*-*	*aph(3’)-lll, aac(6’)-li,* and *aac(6’)-aph(2’’)*	*erm(A), msr(C),* and *erm(B)*	*tet(M)*	*dfrE*	*fexA*	*repUS1* and *repUS15*	*optrA, ermA,* and *fexA on repUS1*
	225A	2239 *	*gyrA_S83I*	*optrA*	*-*	*ant(9)-la* and *aac(6’)-li*	*erm(A)* and *msr(C)*	*tet(L)*	*-*	*fexA*	*repUS43, repUS15, rep22,* and *repUS1*	*tet(L) on repUS43*
	228A	2236 *	*eatA_T450I*	*optrA*	*-*	*ant(6)-la, aph(3’’)-lll,* and *aac(6’)-li*	*msr(C)* and *ermB*	*tet(L)*	*-*	*fexA*	*rep2* and *repUS15*	*-*
	170C	2236 *	*eatA_T450I*	*optrA*	*-*	*ant(6)-la, aph(3’’)-lll, aac(6’)-li,* and *aac(6’)-aph(2’’)*	*erm(A), msr(C),* and *erm(B)*	*tet(L)*	*dfrE*	*fexA*	*rep2* and *repUS15*	*-*
	232A	2238 *		*optrA*	*lnu(B)*	*ant(6)-la, aph(3’)-lll, aac(6’)-li,* and *aac(6’)-aph(2’’)*	*erm(A), erm(B),* and *lsa(E)*	*tet(L)* and *tet(S)*	*dfrG*	*fexA*	*rep22, repUS43,* and *repUS15*	*tet(L) on rep22*

* Fosfomycin: one *E. faecalis* (strain 240B) harbored the *fosB3* resistance gene. * new STs, first reported in this study, that were unavailable in the PubMLST database.

**Table 3:** Virulence gene profiles based on the whole-genome sequencing of the *optrA*-carrying linezolid-resistant *Enterococcus faecalis* strains from retail chicken carcasses.

Strains *	Virulence Genes								
	Sex Pheromones	Adhesion	Invasion	Aggregation	Cytolytic Toxin	Biofilm Formation	Antiphagocytic	Immunity	Protease
171B	*cCF10, cOB1, cad,* and *camE*	*SrtA, ebpA, ebpC,* and *efaAfs*	*hylA*	*-*	*-*	*-*	*tpx*	*ElrA*	*-*
223B	*cCF10, cOB1, cad,* and *camE*	*SrtA, ebpA, ebpC,* and *efaAfs*	*hylA*	*-*	*-*	*-*	*tpx*	*ElrA*	*-*
227B	*cCF10, cOB1, cad,* and *camE*	*SrtA, ebpA, ebpC,* and *efaAfs*	*-*	*-*	*-*	*-*	*tpx*	*ElrA*	*-*
173B	*cCF10, cOB1, cad,* and *camE*	*SrtA, ebpA, ebpC,* and *efaAfs*	*hylB*	*-*	*-*	*fsrB*	*tpx*	*ElrA*	*gelE*
176B	*cCF10, cOB1, cad,* and *camE*	*SrtA, ebpA, ebpC,* and *efaAfs*	*hylB*	*-*	*-*	*fsrB*	*tpx*	*ElrA*	*gelE*
180B	*cCF10, cOB1, cad,* and *camE*	*SrtA, ebpA, ebpC,* and *efaAfs*	*hylA* and *hylB*	*-*	*-*	*fsrB*	*tpx*	*ElrA*	*gelE*
295B	*cCF10, cOB1, cad,* and *camE*	*SrtA, ebpA, ebpC,* and *efaAfs*	*hylA*	*-*	*cylB, cylL,* and *cylM*	*-*	*tpx*	*ElrA*	*-*
279B	*cCF10, cOB1, cad,* and *camE*	*SrtA, ebpA, ebpC,* and *efaAfs*	*hylB*	*-*	*-*	*fsrB*	*tpx*	*ElrA*	*gelE*
302B	*cCF10, cOB1, cad,* and *camE*	*SrtA, ebpA, ebpC,* and *efaAfs*	*hylA*	*-*	*-*	*-*	*tpx*	*ElrA*	*-*
240B	*cCF10, cOB1, cad,* and *camE*	*SrtA, ebpA, ebpC,* and *efaAfs*	*hylA*	*-*	*-*	*-*	*tpx*	*ElrA*	*-*

* Out of the six sequenced *E. faecium* strains, only one virulence (adhesins) gene (*acm*) was identified in one isolate.

## Data Availability

All genome assemblies generated in this study have been deposited in the National Center for Biotechnology Information (NCBI) database and are accessible under the BioProject ID PRJNA850926. The BioSamples accession codes of the studied isolates are: SAMN29206312; SAMN29206313; SAMN29206314; SAMN29206315; SAMN29206316; SAMN29206317; SAMN29206318; SAMN29206319; SAMN29206320; SAMN29206321; SAMN29206322; SAMN29206323; SAMN29206324; SAMN29206325; SAMN29206326; and SAMN29206327.
